# Listeria monocytogenes meningoencephalitis in a patient with Systemic Lupus Erythematosus

**DOI:** 10.1590/2175-8239-JBN-2019-0212

**Published:** 2020-05-11

**Authors:** Maria Eduarda Vilanova da Costa Pereira, Diego Ennes Gonzalez, Fernanda Badiani Roberto, Renato Demarchi Foresto, Gianna Mastroianni Kirsztajn, Marcelino de Souza Durão

**Affiliations:** 1 Universidade Federal de São Paulo, Escola Paulista de Medicina, Departamento de Medicina, Disciplina de Nefrologia, São Paulo, Brasil.

**Keywords:** Listeria monocytogenes, Meningitis, Lupus Erythematosus, Systemics, Listeria monocytogenes, Meningite, Lúpus Eritematoso Sistêmico

## Abstract

**Introduction:**

Infectious complications are common in systemic lupus erythematosus. Although uncommon, central nervous system infections do occur and have significant lethality, with several etiological agents.

**Methods:**

We report on the case of a 29-year-old woman recently diagnosed with systemic lupus erythematosus with hematological, cutaneous, serous and renal manifestations (class IV lupus nephritis), who underwent corticosteroid pulse therapy and mycophenolate induction therapy. After 3 months of evolution, she developed headache and altered mental status. Computed tomography showed an area of hypoattenuation in the left frontal white matter and her cerebrospinal fluid examination showed pleocytosis and hyperproteinorrhachia. Peripheral blood and CSF culture identified Listeria monocytogenes. The patient presented deterioration of her neurological status, requiring invasive mechanical ventilation, monitoring of intracranial pressure and, despite all the intensive support, persisted in a comatose state and developed multiple organ failure, evolving to death due to nosocomial bloodstream infection.

**Discussion:**

Infection from L. monocytogenes usually occurs after eating contaminated food, manifesting itself with diarrhea and, occasionally, invasively, such as neurolisteriosis. Further investigation with CSF analysis and MRI is necessary, and the diagnosis consists of isolating the bacteria in sterile body fluid.

**Conclusion:**

The case presents a patient whose diagnosis of meningoencephalitis became an important differential with neuropsychiatric disorder. The poor outcome reinforces the need to remember this infectious condition as a serious complication in the natural history of SLE.

## Resumo

**Introdução:**

As complicações infecciosas são frequentes no lúpus eritematoso sistêmico. Apesar de incomum, infecções do sistema nervoso central ocorrem e têm significativa letalidade, apresentando diversos agentes etiológicos.

**Métodos:**

Descrevemos aqui o caso de uma mulher de 29 anos recentemente diagnosticada com lúpus eritematoso sistêmico com manifestações hematológica, cutânea, serosa e renal (nefrite lúpica classe IV), submetida a pulsoterapia com corticoide e terapia de indução com micofenolato. Após 3 meses de evolução, apresentou quadro de cefaleia e alteração de estado mental. Tomografia computadorizada evidenciou área de hipoatenuação em substância branca frontal esquerda e exame de líquido cefalorraquidiano mostrava pleocitose e hiperproteinorraquia. Cultura de sangue periférico e do liquor identificaram *Listeria monocytogenes*. Paciente apresentou deterioração do quadro neurológico, necessitando de ventilação mecânica invasiva, monitorização de pressão intracraniana e, apesar de todo o suporte intensivo, persistiu em estado comatoso e disfunção de múltiplos órgãos, evoluindo a óbito por infecção de corrente sanguínea nosocomial.

**Discussão:**

Infecção por *L. monocytogenes* ocorre geralmente após ingestão de alimentos contaminados, manifestando-se por diarreia e, eventualmente, de forma invasiva como a neurolisteriose. Investigação complementar com análise de liquor e ressonância magnética faz-se necessária, sendo o diagnóstico confirmado por isolamento da bactéria em líquido corporal estéril.

**Conclusão:**

O caso representa uma paciente cujo diagnóstico de meningoencefalite tornou-se importante diferencial com atividade de doença neuropsiquiátrica. A evolução insatisfatória reforça a necessidade de se lembrar desta condição infecciosa como complicação grave na história natural do LES.

## Introduction

Systemic Lupus Erythematosus (SLE) is a systemic, inflammatory, autoimmune disease, of multifactorial etiology related to hormonal, genetic and environmental factors. It has multisystemic manifestations, in addition to high morbidity and mortality, mainly due to disease activity or infectious complications.^1^

The high risk of infection in SLE has several causes, including the immune dysfunction inherent to the disease, with the production of autoreactive antibodies and a consequent reduction in immune tolerance, added to clinical factors exemplified by complement deficiency and immunosuppressive treatment.^2^^,^ 3 It represents a frequent complication, present in up to 36% of the cohorts described, in addition to being the cause of death in up to 30% of patients.^4^ Urinary tract and skin are the main sites of community infection, whereas respiratory infections are more frequent in hospitalized patients.^4^

Central nervous system (CNS) infections are uncommon, but have significant lethality, reaching 40%.^5^ The etiological agents vary according to the population studied, with Mycobacterium tuberculosis and Cryptococcus neoformans being the most common. Listeria monocytogenes is less frequently described, but has a high severity.^5^ Possible risk factors for meningoencephalitis are lupus activity, use of high doses of prednisone and hypoalbuminemia.^5^^,^ 6 We describe a case of CNS listeriosis after immunosuppression in a patient with a recent diagnosis of SLE, as well as a literature review on the topic.

## Case report

Female patient, 29 years old, without pathological history, with erythematous lesions in the lower limbs and systemic arterial hypertension, diagnosed seven months before admission. Anti-hypertensive therapy was started by the attending physician, but with little response. She was then admitted to the emergency department complaining of atypical chest pain, the investigation of which revealed cavitary effusions (pleural and pericardial), corroborating suspicion of a systemic disease. The hypothesis of active SLE was suggested, considering the presence of serositis, cutaneous involvement, renal involvement (hematuria and proteinuria) and positivity for the 1: 640 anti-nucleus factor (homogeneous nuclear pattern). Based on clinical and laboratory data, without renal biopsy, we opted for intravenous pulse therapy with methylprednisolone for 3 days, followed by prednisone 1 mg/kg/day orally, in addition to starting mycophenolate mofetil. At that time, serum creatinine was 0.80 mg/dL.

After three months, she returned to the clinic with diarrhea and asthenia, as well as edema of the lower limbs and perineal lesions, characteristic of genital herpes. Laboratory tests showed an increase in serum creatinine (1.50 mg/dL), dysmorphic hematuria of 207,000/mL and 24-hour proteinuria of 5 g, serum albumin of 3.0 g/dL, serum hemoglobin of 6.0 g/dL, without evidence of hemolysis. At that moment, considering the activity score of the SLEDAI-based disease (Systemic Lupus Erythematosus Disease Activity Index), our patient had a score of 20: urinary changes (hematuria with cylinders, proteinuria and pyuria), anti-DNA at high levels, and reduction of serum levels of complement fragments C3 and C4. Results above 8 indicate active disease.

He received red blood cell transfusion and treatment with acyclovir. After clinical improvement, we performed a renal biopsy, showing active/chronic class IV-global lupus nephritis (IV-G A/C) with moderate activity and mild chronicity. In view of the continuous increase in serum creatinine reaching 2.00 mg/dL, a new course of methylprednisolone was indicated for 3 days.

**Figure 1:**
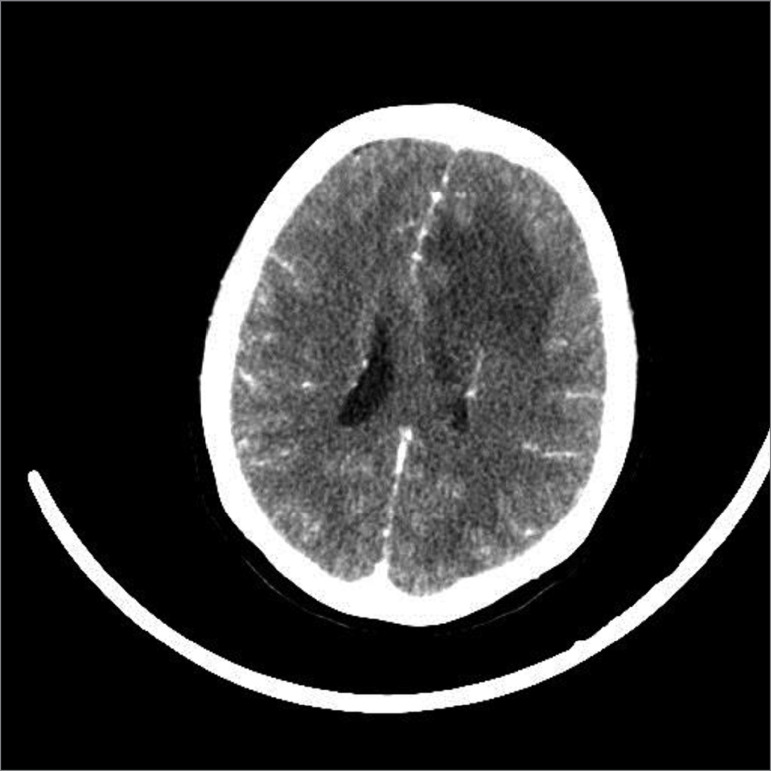
Contrast-enhanced CT scan of the skull. Hypoattenuation area in the left frontal white matter, accentuating the expansive effect on adjacent structures, measuring about 7.0 x 5.0 cm.

Three days after pulse therapy, she developed a headache, vomiting and altered mental status. On physical examination, she had spontaneous eye opening, a reflex of withdrawal from the painful stimulus, but with no verbal response, and was promptly submitted to cranial computed tomography (Figure 1). The Imaging study showed hypodensity in the frontal lobe, compatible with edema, with no evidence of intracranial hypertension. A lumbar puncture was performed, with an opening pressure of 73 cm H_2_O, and the cerebrospinal fluid chemocytological analysis showed: 285 cells per mm^3^ (60% of lymphocytes), hyperproteinorrhachia (112 mg/dL) and hypoglycorrhachia (27 mg/dL). In culture, Listeria monocytogenes grew, as well as in peripheral blood samples. The search for C. neoformans and acid-fast bacillus was negative.

Initially, we chose an empirical treatment with ceftriaxone, vancomycin and acyclovir. After differentiation in culture, the antimicrobial regimen was changed to intravenous ampicillin. The patient’s neurological status deteriorated and she was transferred to the intensive care unit, requiring invasive and hemodynamic ventilatory support, requiring norepinephrine to stabilize blood pressure. External ventricular bypass was performed, maintained for five days, with intracranial pressure monitoring. Concomitantly, she developed azotemia and oliguria, with intermittent hemodialysis beginning with a short-term catheter.

**Figure 2:**
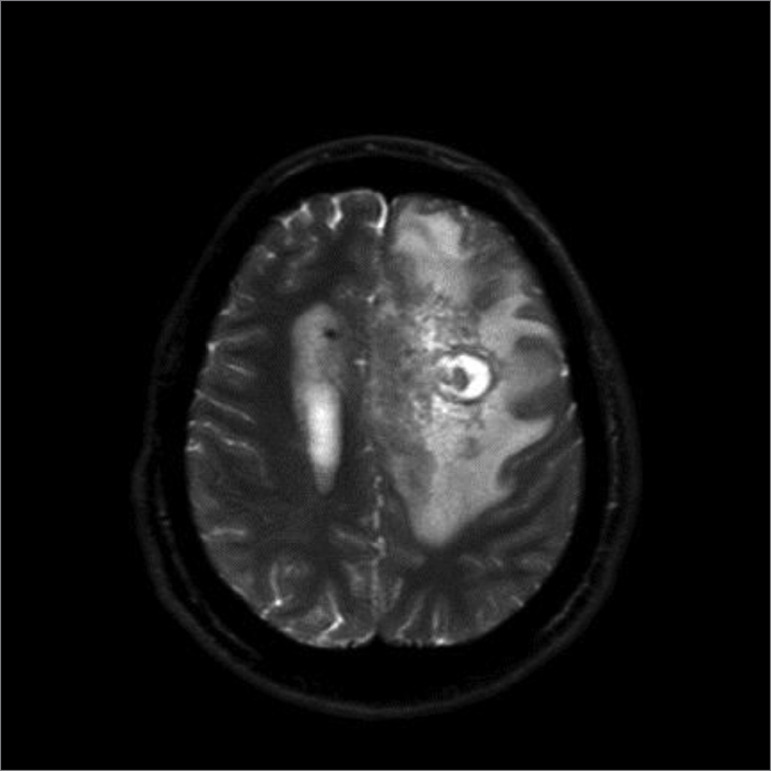
Magnetic resonance imaging of the skull, T2 heavy echo turbo-spin technique. Expansive formation, 3.0 x 3.0 x 3.5 cm, in the left frontal lobe, affecting mainly the upper and middle frontal gyres, with irregular contours, bordered by foci of hemosiderin deposits, with an intense restriction on inside diffusion, compatible with an abscess.

After hemodynamic improvement, we suspended her parenteral sedation and analgesia, but there was no satisfactory neurological evolution. The neurosurgery team’s assessment did not indicate intervention during follow-up. The patient remained in a comatose state for 60 days, with an evolution of the radiological image, whose characteristics matched those of an abscess (Figure 2). The electroencephalographic pattern showed disorganization and slowdown of the basic brain electrical activity, with paradoxical reactivity. In view of the irreversibility of her neurological condition, we opted to start palliative care, and she died after a bloodstream infection.

## Discussion

L. monocytogenes is a Gram-positive bacterium, recognized as a pathogen in the 1970s. The infection is particularly important in the elderly, pregnant women and immunosuppressed patients. It determines mortality close to 30%, which increases with delayed diagnosis.^7^^,^ 8 Its most common forms of manifestation are neurolisteriosis, bacteremia and maternal-neonatal infection.^8^

Listeriosis is a sporadic disease, with outbreaks after eating contaminated food. The bacterium can be found in different environments due to its ability to survive adverse conditions, such as low temperatures.^8^ In the United States, the incidence of confirmed cases is 0.3 per 100,000 people; reaching 1.3 and 3 cases per 100,000 people over 65 and pregnant women, respectively. In Brazil, as it is underdiagnosed and underreported, there are few epidemiological studies available.^9^ In a French retrospective study, which analyzed 1959 cases between the years 2001 to 2008, an increased risk for listeriosis was found in patients with hematological disorders, neoplasms and solid organ transplants. Factors of worse prognosis reported are age over 80 years, positive peripheral blood culture and the presence of comorbidities.^10^

After being ingested, the incubation period of the bacillus varies between 11 and 28 days, when it manifests itself as a self-limited diarrheal syndrome or, in susceptible patients, as an invasive disease, comprising sepsis and, especially, CNS infection of a variable anatomical predilection: meningoencephalitis (the most common), cerebritis, cerebral abscess and rhombencephalitis.^9^^,^ 11 Clinically, the symptoms are variable, with fever and nonspecific alteration in mental status, up to a severe comatose condition. It should be noted that signs of meningeal irritation occur in 40% of the patients.^12^ Laboratory analysis of cerebrospinal fluid shows changes similar to those from other infectious process: pleocytosis (with a predominance of polymorphonuclear or lymphomonuclear), high protein drainage and reduced glycorrhachia. The diagnosis requires isolation of the bacteria in sterile body fluids, such as blood or cerebrospinal fluid,^13^ allowing for the assessment of a susceptibility profile. The examination using the polymerase chain reaction technique has been studied recently, with promising results, as an alternative in cases of high clinical suspicion and without differentiation in cultures.^14^ The best imaging test for studying brain, cortical, trunk and cerebellar is magnetic resonance imaging, showing more specificity in relation to computed tomography.^13^

Despite *in vitro* susceptibility to several classes of antibiotics, there is still no ideal treatment for listeriosis, since the effectiveness of antibiotic therapy reaches approximately 70%. The CNS, making it difficult for the medication to penetrate.8, 15, 16, can list multiple possible causes, such as the characteristic intracellular location of L. monocytogenes and its tropism

Penicillin represents the gold standard therapy, being the most used in the treatment of infection. Ampicillin stands out, which must be prescribed in high dose (9 g/day) for at least 21 days, in cases of CNS involvement,^17^^,^ 18 reaching 6 weeks, in cases of brain abscess.^12^ In patients allergic to penicillin, treatment with Sulfamethoxazole-Trimethoprim is recommended. Other antibiotics with documented *in vitro* action against L. monocytogenes are quinolones, vancomycin, linezolid and meropenem; however, with limited evidence, mainly regarding its effectiveness in neurological disease. Combined ampicillin therapy associated with gentamicin, aiming at synergism, is described, but with conflicting results, which can be explained by the low penetration of this drug in cerebrospinal fluid.^17^^-^ 19 The use of corticosteroids for patients with listeria meningoencephalitis is related to controversial outcomes,^10^^,^ 20 there is not enough evidence to indicate their prescription.^18^

In the case reported, despite the rapid diagnosis and the initiation of adequate therapy, the neurological damage was irreversible and the evolution was unfavorable. In addition to the risk factors already discussed, SLE activity has been associated with the risk of infection in this population, in a way directly proportional to the gradation of the activity index according to the SLEDAI criteria. Upon admission, our patient had a SLEDAI of 20, characterizing high disease activity.^3^ Therefore, it becomes a challenge to differentiate neurological symptoms secondary to the infection of a neurological condition that corresponds to the underlying disease (neuropsychiatric lupus), since symptoms such as headache, seizure and fever occur in both entities.

## Conclusion

Despite being an uncommon cause of CNS infection in SLE patients, L. monocytogenes needs to be remembered as an etiological agent, since this population is at risk due to immunosuppression and because it is a highly lethal disease. The unusual clinical picture can delay a possible diagnosis, and consequently further worsen the evolution.
